# Immune cells dying from ferroptosis: mechanisms and therapeutic opportunities

**DOI:** 10.1038/s41419-025-08204-9

**Published:** 2025-12-01

**Authors:** Ji Liu, Rufang Dong, Bingqing Yuan, Yitong Xie, Ziqin Feng, Shengtao Zhou, Fujuan Luan, Yanjun Chen

**Affiliations:** Department of Gastroenterology, The First Affiliated Hospital of Soochow University, Suzhou, 215000 Jiangsu Province China

**Keywords:** Immune cell death, Cell death

## Abstract

This review explores the intricate regulation of ferroptosis through diverse pathways and related metabolism, focusing on the mechanisms of ferroptosis in diverse immune cells, including granulocytes, macrophages, dendritic cells, T/B lymphocytes, natural killer/ innate lymphoid cells, under conditions of iron metabolism imbalance and lipid peroxidation accumulation, highlighting their pivotal roles in the dynamic regulation of immune microenvironments. Furthermore, the review summarizes the therapeutic potential of targeting ferroptosis pathways in tumor immunity, pathogen infections, inflammation, and autoimmune diseases. It systematically compiles recent advances in ferroptosis-related drugs and small molecule inhibitors, while proposing novel therapeutic strategies through intervention in ferroptosis-immune interactions for immune-related disorders. Additionally, this review summarizes the mechanisms of epigenetic regulation of ferroptosis. As a core enzyme in epigenetic regulation, Enhancer of zeste homolog 2 (EZH2) serves as a pivotal node linking epigenetic regulation, tumorigenesis, immune control, and ferroptosis, providing novel therapeutic insights for anti-tumor immunity.

## Facts

- Ferroptosis is regulated by multiple pathways and is intricately interconnected with metabolic processes.
- The mechanisms of ferroptosis in different immune cells (such as granulocytes, macrophages, dendritic cells, T/B lymphocytes, natural killer cells/intrinsically lymphoid cells, etc.) under conditions of iron metabolism imbalance and lipid peroxidation accumulation are the focus of future research.
- Targeting ferroptosis pathways holds potential for the treatment of tumor immunity, pathogen infections, inflammation, and autoimmune diseases. The development of drugs and small molecule inhibitors targeting ferroptosis-related targets is a future trend.
- Enhancer of zeste homolog 2 (EZH2) serves as a pivotal enzyme in epigenetic regulation linking epigenetic regulation, tumorigenesis, immune control, and ferroptosis.

## Pathway and Metabolism in Ferroptosis

In 1876, Fenton first reported the reaction between iron salts and hydrogen peroxide to generate hydroxyl radicals, proposing what is now known as the Fenton reaction, named after him [1]. Ferroptosis is a form of cell death driven by iron-dependent lipid peroxidation, identified and named a decade ago as a distinct phenomenon. It is implicated in a wide range of biological contexts, from development to aging, immunity, and cancer, and is associated with numerous diseases. Ferroptosis typically exhibits necrotic death-like morphological changes, with ultrastructural abnormalities often observed in mitochondria. It involves various biochemical metabolic processes, including iron metabolism, lipid metabolism, and amino acid metabolism [1].

Previous studies suggested that ferroptosis does not cause plasma membrane rupture, with the membrane remaining intact. However, recent findings indicate that membrane rupture does occur during ferroptosis, accompanied by increased cytoplasmic Calcium ion (Ca²⁺) levels before cell swelling [2]. Riegman et al. discovered that ferroptosis can propagate the “cell swelling effect” within a cell population [3]. Bartosz Wiernicki proposed an induction model for ferroptosis, dividing the process into three stages: an “initial” phase associated with lipid peroxidation, an “intermediate” phase marked by Adenosine Triphosphate release, and a “terminal” phase identified by High Mobility Group Box 1 **(**HMGB1) release and loss of plasma membrane integrity [4].

### Regulation of ferroptosis through diverse pathways

#### The system Xc^-^/ GSH/ GPX4 axis

**Fig. 1:**
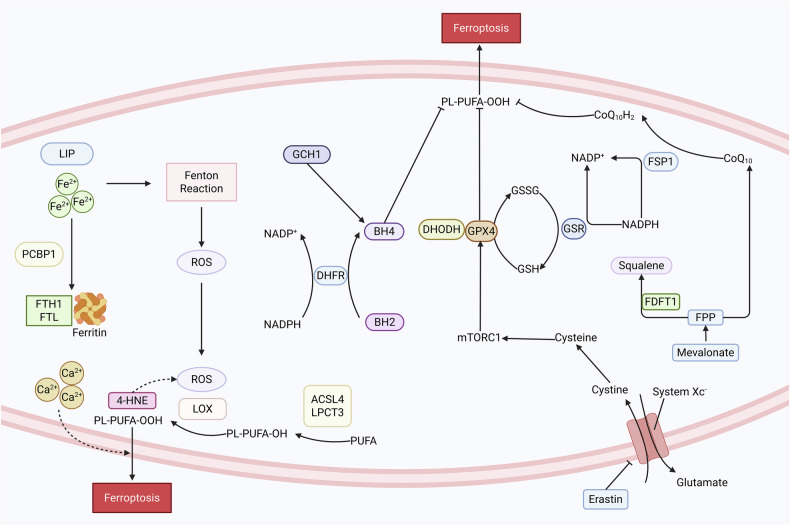
Regulation of various pathways in ferroptosis. The System Xc^-^/ Glutathione (GSH)/ Glutathione Peroxidase 4 (GPX4) axis, characterized by elevated GSH and GPX4 expression, represents the classical pathway of ferroptosis. Inhibition of System Xc⁻ e.g. by Erastin or cysteine depletion leads to GSH exhaustion and GPX4 inactivation, triggering lipid peroxidation and ferroptosis. Ferroptosis suppressor protein 1(FSP1), an Nicotinamide Adenine Dinucleotide Phosphate (NADPH)-selective enzyme, suppresses ferroptosis by oxidizing NADPH. FSP1 targets Coenzyme Q10(CoQ10) in the cell membrane to trap lipid peroxidation radicals, thereby inhibiting lipid peroxidation. In the GTP cyclohydrolase 1(GCH1)/ Tetrahydrobiopterin (BH4) system, Dihydrofolate Reductase (DHFR) also utilizes NADPH to promote BH4 biosynthesis. BH4 acts as a radical-trapping antioxidant that inhibits ferroptosis. Dihydroorotate Dehydrogenase (DHODH) cooperates with GPX4 to suppress ferroptosis in the mitochondrial membrane, and DHODH deficiency promotes ferroptosis. Squalene, a lipophilic metabolite, has gained attention for its role in ferroptosis, as it protects cells from ferroptosis through the FSP1-CoQ10 axis. Created in https://BioRender.com.

The System Xc^-^ (the cystine/glutamate antiporter system) / Glutathione (GSH) / Glutathione Peroxidase 4 (GPX4) axis, characterized by elevated GSH and GPX4 expression, represents the canonical pathway of ferroptosis. This system exhibits high specificity for cystine and glutamate, facilitating the uptake of cystine into cells while exporting glutamate extracellularly. Intracellular cystine is subsequently reduced to cysteine, which participates in GSH synthesis. Glutathione-disulfide reductase (GSR) utilizes Nicotinamide Adenine Dinucleotide Phosphate (NADPH) as a cofactor to generate reduced GSH. GPX4 functions as an intracellular enzyme that reduces lipid hydroperoxides and relies on reduced GSH to eliminate lipid peroxides. When GSH is depleted or GPX4 is inactivated, lipid peroxides accumulate, ultimately triggering ferroptosis (Fig. 1).

#### FSP1/ CoQ_10_/NADPH axis

Ferroptosis suppressor protein 1 (FSP1) inhibits lipid peroxidation and ferroptosis independently of the GPX4 and GSH pathways. As an NADPH-selective enzyme, FSP1 suppresses ferroptosis by oxidizing NADPH. It localizes to the plasma membrane and targets Coenzyme Q10 (CoQ10), catalyzing the reduction of CoQ10 or vitamin K. This reduction process enables the scavenging of lipid peroxidation radicals, thereby inhibiting lipid peroxidation (Fig. 1).

#### The GCH1/BH4/DHFR axis

Tetrahydrobiopterin (BH4) plays multifaceted roles in regulating oxidative stress and inflammation. GTP cyclohydrolase 1 (GCH1) serves as the first rate-limiting enzyme in BH4 biosynthesis. BH4 functions as a radical-trapping antioxidant capable of inhibiting ferroptosis. Research demonstrates that both GCH1 and BH4 protect cells from lipid peroxidation damage during ferroptosis induction. Within the GCH1/BH4 system, dihydrofolate reductase (DHFR) further utilizes NADPH to facilitate BH4 production [5] **(**Fig. 1**)**.

#### Novel pathways

Dihydroorotate Dehydrogenase (DHODH) is a mitochondrial enzyme that generates ubiquinol (reduced coenzyme Q10). Mao et al. identified that DHODH cooperates with GPX4 to suppress ferroptosis in mitochondrial membranes. Loss of DHODH promotes ferroptosis, particularly in cancer cells with low GPX4 expression levels [6]. Pleckstrin homology-like domain family A member 2 operates independently of both Acyl-CoA Synthetase Long-chain family member 4 (ACSL4) and conventional ferroptosis inducers [7]. Squalene, a lipophilic metabolite, has recently emerged as a regulator of ferroptosis. Cholesterol modulates ferroptosis susceptibility by altering metabolic flux in the mevalonate pathway through targeted degradation of squalene epoxidase (SQLE), the rate-limiting enzyme in cholesterol biosynthesis. This degradation leads to accumulation of both CoQ10 and squalene, which collectively confer ferroptosis resistance via the FSP1-CoQ10 antioxidant axis [8] (Fig. 1).

### Ferroptosis and related metabolism

#### Ferroptosis and iron metabolism

**Fig. 2:**
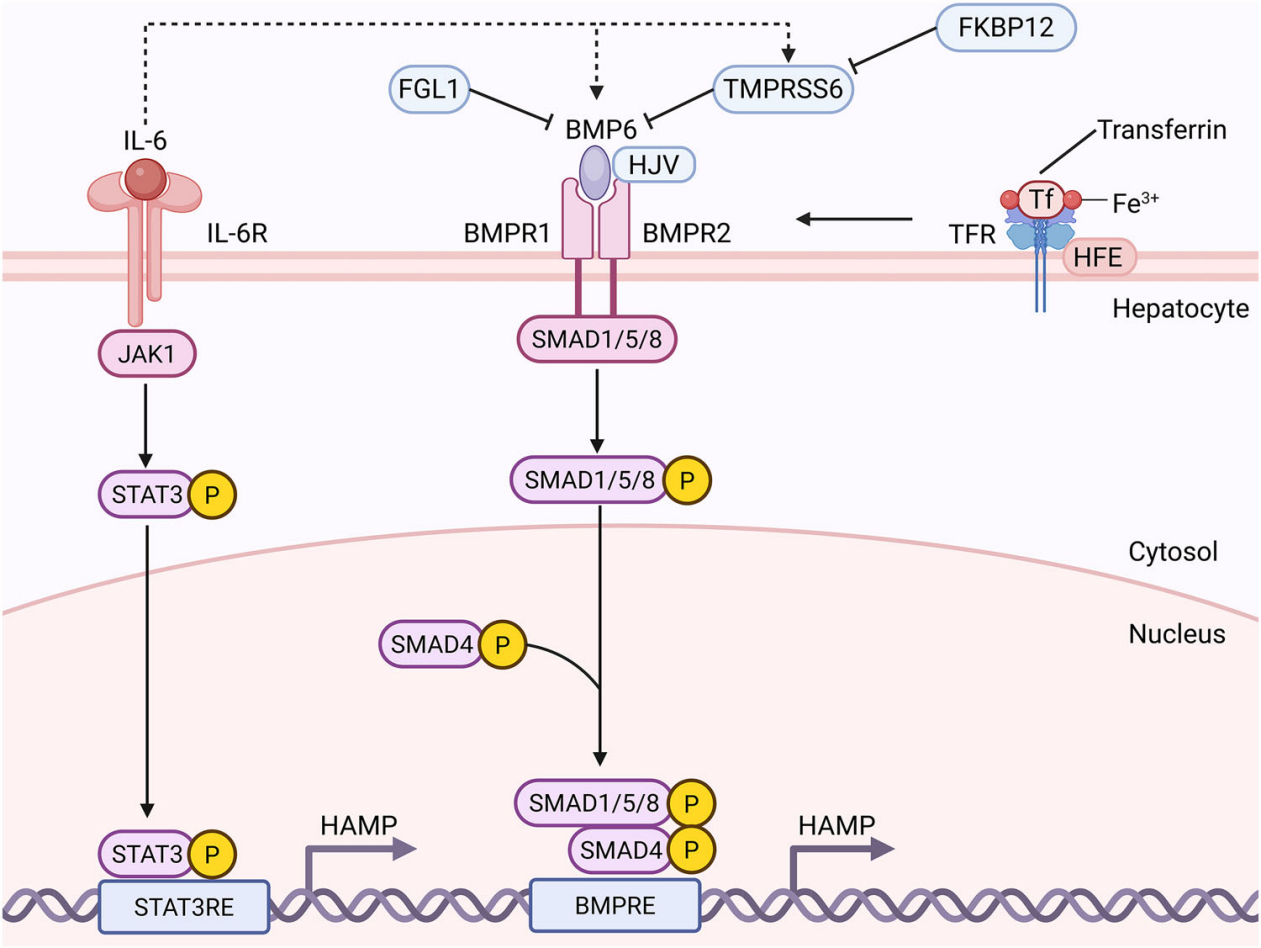
The primary pathways related to the encoding of the *HAMP* gene. The Small Mothers Against Decapentaplegic (SMAD) pathway is activated through bone morphogenetic protein 6 (BMP6) and other BMPs binding to heterodimeric BMP receptor (BMPR) I/II [10]. Iron triggers BMP6 induction via multiple mechanisms: autonomous activation through Nuclear factor erythroid 2-related factor 2 (Nrf2)-mediated oxidative stress sensing, and transcriptional regulation through E26 Transformation-Specific 1 (ETS1) and p38 mitogen-activated protein kinase pathway [20]. BMP6 and Hemojuvelin (HJV) cooperatively activate BMPR I/II complex, promoting phosphorylation of downstream BMP mediators like SMAD1/5/8. The phosphorylated SMAD1/5/8 forms an active transcriptional complex with cytoplasmic SMAD4, which translocates into the nucleus. This complex binds to BMP response elements (BMP RE) and subsequently activates HAMP transcription [21, 22]. The hepatokine fibrinogen-like 1 (FGL1) inhibits the canonical BMP-SMAD signaling cascade controlling hepcidin transcription by directly binding to BMP6 [23]. Although endothelial-derived BMP6 and BMP2 ligands play crucial roles as endogenous hepcidin regulators, iron homeostasis and erythropoiesis-driven hepcidin regulation persist in mice deficient in one or both ligands. BMP5 exhibits functional compensation in iron regulation under BMP6-deficient conditions [24]. MT-2/TMPRSS6, widely expressed in the liver, acts as a BMP coreceptor through HJV to enhance signaling. Conversely, TMPRSS6 suppresses this pathway via HJV cleavage, forming a negative feedback loop [10]. Recent studies demonstrate that downregulation of FKBP12 in hepatocytes promotes TMPRSS6 expression, thereby counteracting ALK2-mediated pathway activation. This reveals functional crosstalk between FKBP12 and TMPRSS6 in controlling hepcidin transcription [25]. Interleukin-6 (IL-6), as a pro-inflammatory cytokine, can bind to its receptor and activate the Janus kinase(JAK)/ Signal Transducer and Activator of Transcription 3 (STAT 3) signaling pathway, leading to STAT3 phosphorylation and nuclear translocation. This process enhances the transcriptional activity of the HAMP gene promoter region, thereby promoting hepcidin synthesis [26, 27]. IL-6 regulates hepcidin expression through the BMP/SMAD pathway by altering the expression levels of BMP6, TMPRSS6, and Transferrin receptor 2(TfR2) under both normal and inflammatory conditions. Created in https://BioRender.com.

Iron metabolism, involving absorption, transport, storage, utilization, and excretion in organs like the intestine, liver, and spleen, is highly regulated. Its dysregulation is linked to diseases such as iron-deficiency anemia and hereditary hemochromatosis [9, 10]. Iron disorders also impact chronic inflammatory diseases and cancer [11–15]. Cellular senescence and fibrosis are also associated with iron accumulation [16]. In healthy organisms, iron levels in plasma and storage sites like hepatocytes and spleen are tightly regulated by hepcidin, a liver hormone encoded by Hepcidin Antimicrobial Peptide (HAMP) [17]. The Hepcidin-Ferroportin (FPN) axis controls extracellular iron homeostasis, with hepcidin deficiency causing hereditary hemochromatosis [9, 18]. The Bone Morphogenetic Protein (BMP)/ Small Mothers Against Decapentaplegic (SMAD) and Interleukin (IL)-6/ Signal Transducer and Activator of Transcription (STAT) pathways regulate HAMP transcription, maintaining iron homeostasis and influencing diseases. Transferrin Receptor (TfR) 1, interacting with Homeostatic Iron Regulator (HFE), also modulates hepcidin production [19–27] (Fig. 2).

**Fig. 3:**
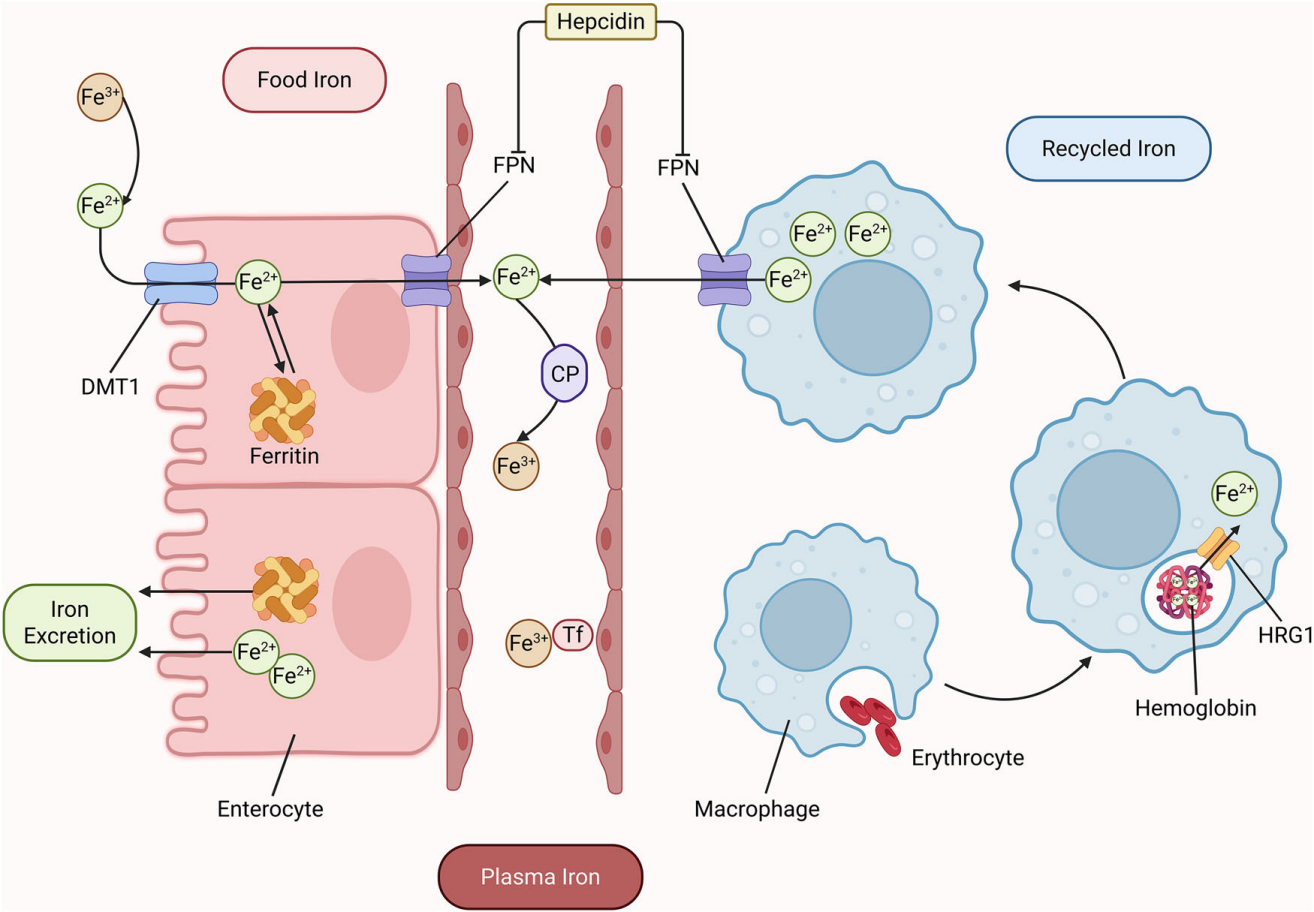
The processes of iron absorption, transport, recycling, and excretion. When systemic iron levels rise, hepcidin degrades Ferroportin (FPN), reducing iron influx into plasma and inhibiting absorption. Conversely, when iron levels drop, hepcidin production decreases, allowing iron absorption to resume and plasma iron levels to rise [10]. Dietary iron primarily exists as Fe³⁺, which cannot be directly absorbed until reduced to Ferrous ion (Fe²⁺) in the duodenum and jejunum. Following absorption by intestinal epithelial cells, most iron binds to heme for incorporation into various biochemical processes as “functional iron”. The remainder enters circulation via basolateral FPN, where it is oxidized to Fe³⁺ by ferroxidase assistance. Plasma iron is transported as Fe³⁺ bound to Transferrin, which delivers it to target organs via systemic circulation. Cellular iron uptake occurs through Transferrin Receptor (TfR) -mediated endocytosis. Within endosomes, Fe³⁺ is reduced to Fe²⁺ by Six Transmembrane Epithelial Antigen of Prostate 3 (STEAP3) and exported to cytoplasm via Divalent Metal Transporter 1 (DMT1) for mitochondrial utilization. A portion of bodily iron constitutes “storage iron” that maintains iron supply and hemoglobin stability. Fe²⁺ is stored in labile iron pools or ferritin complexes composed of Ferritin Heavy Chain 1 (FTH1) and Ferritin Light Chain (FTL). Iron is predominantly stored as ferritin and hemosiderin in mononuclear macrophages of bone marrow, liver, and spleen. Under iron homeostasis, daily exchange between storage pools remains minimal. Hepatic, splenic, and bone marrow macrophages phagocytose senescent erythrocytes. Following erythrocyte degradation in phagolysosomes, hemoglobin-derived heme iron is transported to cytoplasm via Heme Transporter Solute Carrier Family 48 Member 1 (HRG1) for ferritin storage or recirculation. Notably, mammals lack active iron excretion mechanisms. Systemic iron balance is maintained through absorption regulation, with daily losses occurring mainly via intestinal mucosal cell shedding and biliary excretion. Minimal iron is lost through genitourinary excretion, skin desquamation, and sweat. Created in https://BioRender.com.

FPN on duodenal enterocytes mediates iron uptake, while FPN-expressing macrophages regulating its export to plasma. FPN also maintains local iron homeostasis in cells like cardiomyocytes [28]. Ferric ion(Fe³⁺) binds to transferrin, is internalized via TfR-mediated endocytosis, reduced to Ferrous ion (Fe²⁺) by Six-Transmembrane Epithelial Antigen of the Prostate 3 (STEAP3), and released into the cytoplasm by divalent metal transporter 1 (DMT1) for mitochondrial use. Iron excretion mainly occurs through shedding of intestinal mucosal cells (Fig. 3). Grab, highly expressed in differentiated erythroblasts, regulates transferrin cycling and iron metabolism; its deficiency impairs TfR recycling and iron uptake [29]. In iron metabolism pathways, cellular iron storage proteins such as ferritin can protect cells from ferroptosis. For instance, mitochondrial ferritin and Ferritin Heavy Chain (FTH) jointly protect macrophages from RAS-selective lethal 3 (RSL3)-induced ferroptosis under hypoxic conditions [30]. Poly(rC)-binding protein 1, acts as a cytosolic iron chaperone in mammalian cells, binding Fe²⁺ and delivering it to ferritin, thereby limiting cytoplasmic iron toxicity, preventing ferroptosis and lipid peroxidation [31].

#### Ferroptosis and lipid metabolism

Fatty acids are categorized into saturated fatty acids, monounsaturated fatty acids, and polyunsaturated fatty acids (PUFAs). Under the catalysis of Reactive Oxygen Species (ROS) generated by lipoxygenases (LOXs) or the Fenton reaction, PL-PUFA-OH is further oxidized into Phospholipid Polyunsaturated Fatty Acid Hydroperoxide (PL-PUFA-OOH). PUFAs are key drivers of ferroptosis. To exert their pro-ferroptotic effects, PUFAs must first be activated and incorporated into membrane lipids such as phospholipids. Their peroxidation products include Lipid Hydroperoxides (LOOHs) and reactive aldehydes (e.g., malondialdehyde and 4-hydroxynonenal, 4-HNE). 4-HNE can induce ROS production, and excessive ROS leads to oxidative damage in lipid bilayers, triggering ferroptosis. Therefore, 4-HNE serves as a biomarker of ferroptosis. During myocardial infarction, 4-HNE accumulates alongside a high incidence of myocardial ferroptosis. The upregulation of ovarian tumor deubiquitinase 5 deubiquitinates and stabilizes GPX4, reversing 4-HNE-induced ferroptosis, revealing the mechanistic role of 4-HNE in GPX4-dependent ferroptosis [32]. Additionally, ACSL4 and Lysophosphatidylcholine Acyltransferase 3 (LPCAT3) are key lipid drivers of ferroptosis. LPCAT3 cooperates with ACSL4 and Yes-associated protein (YAP) to determine ferroptosis susceptibility [33]. Recent studies show that Protein Kinase C beta II (PKCβII) senses initial lipid peroxidation events, phosphorylates ACSL4, and drives the activation of ACSL4, promoting PUFA incorporation into phospholipids and subsequent cell death [34].

Pooranee et al. demonstrated that differences in PUFA-PL content lead to varying sensitivity to ferroptosis among immune cells. For example, the low PUFA-PL content in activated neutrophils enhances their resistance to ferroptosis [35]. PUFAs can trigger a cytokine response in intestinal epithelial cells (IECs), which is normally restrained by GPX4. In mice with IEC-specific knockout of GPX4, consumption of a PUFA-enriched diet induces focal granuloma-like neutrophilic enteritis [36].

#### Ferroptosis and amino acid metabolism

Amino acid metabolism provides the fundamental basis for ferroptosis. GSH, a crucial tripeptide composed of glutamate, cysteine, and glycine, serves as the primary intracellular antioxidant and detoxifying agent, playing a pivotal role in maintaining cellular redox balance, detoxification, and immune function. Cystine and glutamate regulate intracellular GSH synthesis via System Xc⁻ (SLC3A2 and SLC7A11), thereby modulating susceptibility to ferroptosis.

In 1955, Harry Eagle demonstrated that cysteine is essential for the survival and proliferation of mouse fibroblast L-cells and HeLa cell lines [37]. Jerry Mitchell later discovered that GSH protects against acetaminophen-induced hepatic necrosis in rats [38]. Shiro Bannai reported that cystine deprivation leads to cell death associated with GSH depletion, which can be prevented by vitamin E treatment [39]. Cyst(e)ine activates the mechanistic/mammalian target of rapamycin complex 1 (mTORC1), promoting GPX4 synthesis [40].

Methionine contributes to GSH synthesis via the transsulfuration pathway by converting to cysteine. In both murine and human glioma cell lines, cysteine and methionine deprivation synergizes with the GPX4 inhibitor RSL3 to enhance ferroptosis and lipid peroxidation [41]. Inhibition of protein arginine methyltransferase 5 suppresses GPX4 methylation, reduces GPX4 levels, and increases sensitivity to ferroptosis inducers [42]. Additionally, Hisakatsu Sone et al. found that cisplatin-induced microRNA-429-3p suppresses branched-chain amino acids catabolism in proximal tubules, leading to ferroptosis [43].

## Ferroptosis in immune cells

Immune cells maintain organismal homeostasis through diverse mechanisms, including direct cell-cell interactions, cytokine secretion, and antibody production. Under pathological conditions such as infections, autoimmune diseases, or cancer, immune cell function may become dysregulated, leading to impaired immune responses. Recent studies have identified ferroptosis in immune cells as a critical regulator of immune responses and disease pathogenesis. Below we systematically examine ferroptosis across different immune cell subtypes (Table 1).

**Table 1:** Ferroptosis in immune cells.

Immune Cells	Related Diseases	Key regulatory molecules and associated mechanisms	Ferroptosis
Neutrophil	Brain inflammation	Fluoride induces calcium dyshomeostasis in neutrophils, leading to calcium channel activation and subsequent opening of L-type calcium channels, triggering neutrophil ferroptosis and NETosis (NET release) [45].	Promotion
Neutrophil	Gastric cancer	Spontaneous ferroptosis releases oxidized lipids that constrain T cell activity [46]	Promotion
Neutrophil	Hepatoblastoma	CXCR4 is highly expressed in TIN and releases oxidized lipids that suppress T cell activity [47]	Promotion
Neutrophil	Breast cancer	The GM-CSF–JAK/STAT5–C/EBPβ axis activates ACOD1 to produce itaconate, mediating Nrf2-dependent ferroptosis resistance [48]	Inhibition
Neutrophil	Breast cancer	IL-1β + CXCL3 + CD4 + T cells mediate neutrophil ferroptosis [49]	Promotion
Neutrophil	Systemic Lupus Erythematosus	Autoantibodies and IFN-α present in serum enhance the binding of transcriptional repressor CREMα to the GPX4 promoter [50].	Promotion
Neutrophil	Allergic contact dermatitis (ACD)	DCNB-induced spontaneous neutrophil ferroptosis in ACD mouse model [51]	Promotion
Neutrophil	Post-traumatic brain injury	FOXO1- TfR axis drives neutrophil ferroptosis [52]	Promotion
Neutrophil	Sepsis-associated acute lung injury	Serum response factor-mediated MLCK transcription protects neutrophils from ferroptosis in myocardial tissue [53]	Inhibition
Eosinophil	Asthma	CREB knockdown potentiates dexamethasone-induced eosinophil ferroptosis [55]	Promotion
Macrophage	Hemochromatosis	Ferric citrate overload induces ferroptosis in mouse bone marrow-derived macrophages via SLC7A11 upregulation, ROS accumulation [59]	Promotion
Macrophage	Transfusion model	HO-1 upregulation fails to counteract ferroptosis in macrophages after massive erythrophagocytosis [60]	Promotion
Macrophage	Systemic sclerosis	ACSL4-mediated inflammatory macrophage ferroptosis exacerbates fibrosis [61]	Promotion
Macrophage	Ulcerative colitis	ERK-cPLA2-ACSL4-mediated Arachidonic Acid metabolic activation underlies M2 Macrophage ferroptosis vulnerability [63]	Promotion
Macrophage	Necrotic enteritis	Fe_3_O_4_ nanoparticles induce M1-polarizing shift and trigger macrophage ferroptosis after 48-hour exposure [65]	Promotion
Macrophage	Atherosclerosis	Micheliolide activates the Nrf2 pathway, inhibits macrophage ferroptosis [66]	Inhibition
Macrophage	Atherosclerosis	Tricetin activates the Nrf2 pathway, inhibits macrophage ferroptosis and oxidative stress [67]	Inhibition
Macrophage	Atherosclerosis	Cigarette tar triggers macrophage ferroptosis via the NF-κB-activated hepcidin/FPN/SLC7A11 pathway [68]	Promotion
Macrophage	Atherosclerosis	Heme-induced macrophage ferroptosis promotes p38 pathway activation and MMP2/9 overexpression [62]	Promotion
Macrophage	Cardiac remodeling	Knockout of IL-23p19 reduces macrophage ferroptosis and improves cardiac remodeling [69]	Inhibition
Macrophage	Lung cancer	Dihydroartemisinin upregulates TfR1, increasing intracellular iron levels, and inhibits GPX4, inducing tumor-associated macrophage ferroptosis [70]	Promotion
Macrophage	Hepatocellular carcinoma	Inhibition of APOC1 reverses M2-polarized macrophages to the M1 phenotype via ferroptosis [71]	Promotion
Macrophage	Hepatocellular carcinoma	xCT-mediated macrophage ferroptosis significantly upregulates PD-L1 expression in macrophages [72]	Promotion
Macrophage	Hepatocellular carcinoma	miR-142-3p promotes M1 macrophage ferroptosis in HBV infection via SLC3A2 [73]	Promotion
Macrophage	Rheumatoid arthritis	The HMGB1/TLR4/STAT3 axis plays a crucial role in M2 macrophage ferroptosis and exacerbation of synovial inflammation [74]	Promotion
Macrophage	Necrotic enteritis	Clostridium perfringens beta-1 toxin induces macrophage ferroptosis through sustained increases in intracellular calpain and oxidative stress [65]	Promotion
Macrophage	Silicosis	Silica induces macrophage ferroptosis in mice via Wnt5a/Ca2+ signaling activation, endoplasmic reticulum stress, and mitochondrial redox imbalance [75]	Promotion
Macrophage	Spinal cord injury	CA-074-me inhibits cathepsin B, alleviating macrophage ferroptosis [76]	Inhibition
Macrophage	Acute lung injury caused by sepsis	Uridine suppresses macrophage ferroptosis by activating the Nrf2 signaling pathway [77]	Inhibition
Macrophage	Acute lung injury	Through activation of p38 MAPK and STAT6 signaling, TREM2 is downregulated in LPS-treated macrophages and in acute lung injury mice, inhibiting macrophage ferroptosis [78]	Inhibition
Macrophage	Liver injury	HMOX1-mediated ferroptosis in Clec4F + /CD206+ Kupffer cells triggers NLRP3 inflammasome activation and IL-1β release, exacerbating heat stroke-induced liver injury [79]	Promotion
Dendritic cell	Glioblastoma	Rab27a downregulation suppresses glioblastoma-derived exosome secretion, attenuating lipid accumulation and peroxidation in infiltrating brain dendritic cells [82]	Inhibition
Dendritic cell	Tumor microenvironment	PD-L1 deficiency enhances lipid peroxidation and dendritic cell ferroptosis, compromising antitumor immunity [83]	Promotion
Dendritic cell	Tumor microenvironment	The activation of endoplasmic reticulum stress response factor X-box binding protein 1 in dendritic cells suppresses antitumor immunity by promoting abnormal lipid accumulation [84]	Promotion
CD8 + T cell	Tumor microenvironment	CD36 mediates fatty acid uptake by tumor-infiltrating CD8 + T cells, inducing lipid peroxidation and ferroptosis [85, 86]	Promotion
CD8 + T cell	Tumor microenvironment	Cystine deprivation triggers CD36-mediated ferroptosis and dysfunction of tumor-infiltrating CD8 + T cells [87]	Promotion
CD8 + T cell	Tumor microenvironment	Anti-CD274 immunotherapy induces IFN-γ release from CD8 + T cells, suppressing SLC7A11 and promoting ferroptosis [88]	Promotion
CD8 + T cell	Tumor microenvironment	DEPDC5-deficient CD8 + T cells exhibit spontaneous ferroptosis due to MTORC1-driven ATF4 upregulation [92]	Promotion
CD8 + T cell	Tumor microenvironment	PD-1 signaling suppresses phospholipid phosphatase 1 expression, exacerbating CD8 + T cell ferroptosis [93]	Promotion
CD4 + T cell	Infection with acute lymphocytic choroid meningitis virus or Leishmania	Selenium supplementation enhances GPX4 expression in follicular helper T cells, reducing ferroptosis susceptibility [94]	Inhibition
CD4 + T cell	Post-traumatic sepsis	CD4 + T cell ferroptosis is mediated through suppression of the xCT-GSH-GPX4 axis [97]	Promotion
CD4 + T cell	Iron overload	Iron-overloaded CD4 + T cells exhibit defective mitochondrial iron regulation, spontaneous hyperproliferation, and mitochondrial hyperactivity [95]	Promotion
CD4 + T cell	Acute type A aortic dissection	Elevated CD36 in CD4 + T cells exacerbates palmitate-induced ferroptosis and hypofunction [96]	Inhibition
Regulatory T cell	High-fat diet related colitis	High-fat diet rich in PUFAs primes Tregs for lipid peroxidation and ferroptosis vulnerability [98]	Promotion
Naïve T cell	Autoinflammatory disease	Trappc1 intrinsically suppresses naïve T cell ferroptosis to maintain T cell homeostasis [100]	Inhibition
B lymphocyte	Systemic Lupus Erythematosus	B Cell ferroptosis regulate its differentiation and plasma cell formation [102]	Promotion
Natural killer	Tumor microenvironment	In the tumor microenvironment, NK cells efficiently produce IFN-γ [105]. IFN-γ-producing immune cells can trigger ferroptosis in lipid-rich microenvironments [89].	Promotion
Innate Lymphoid Cell	Intestinal inflammation	GPX4-mediated ferroptosis in NKp46 + ILC3s is regulated by the LCN2-p38-ATF4-xCT signaling axis [107]	Promotion

### Granulocyte

Neutrophils are key cells in combating bacterial and fungal infections. Neutrophil death has significant physiological implications, with different death pathways depending on their interactions with microbes and the external environment, leading to diverse immune outcomes [44]. Although research into ferroptosis has flourished, the understanding of its impact on neutrophils is still in its infancy.

**Fig. 4:**
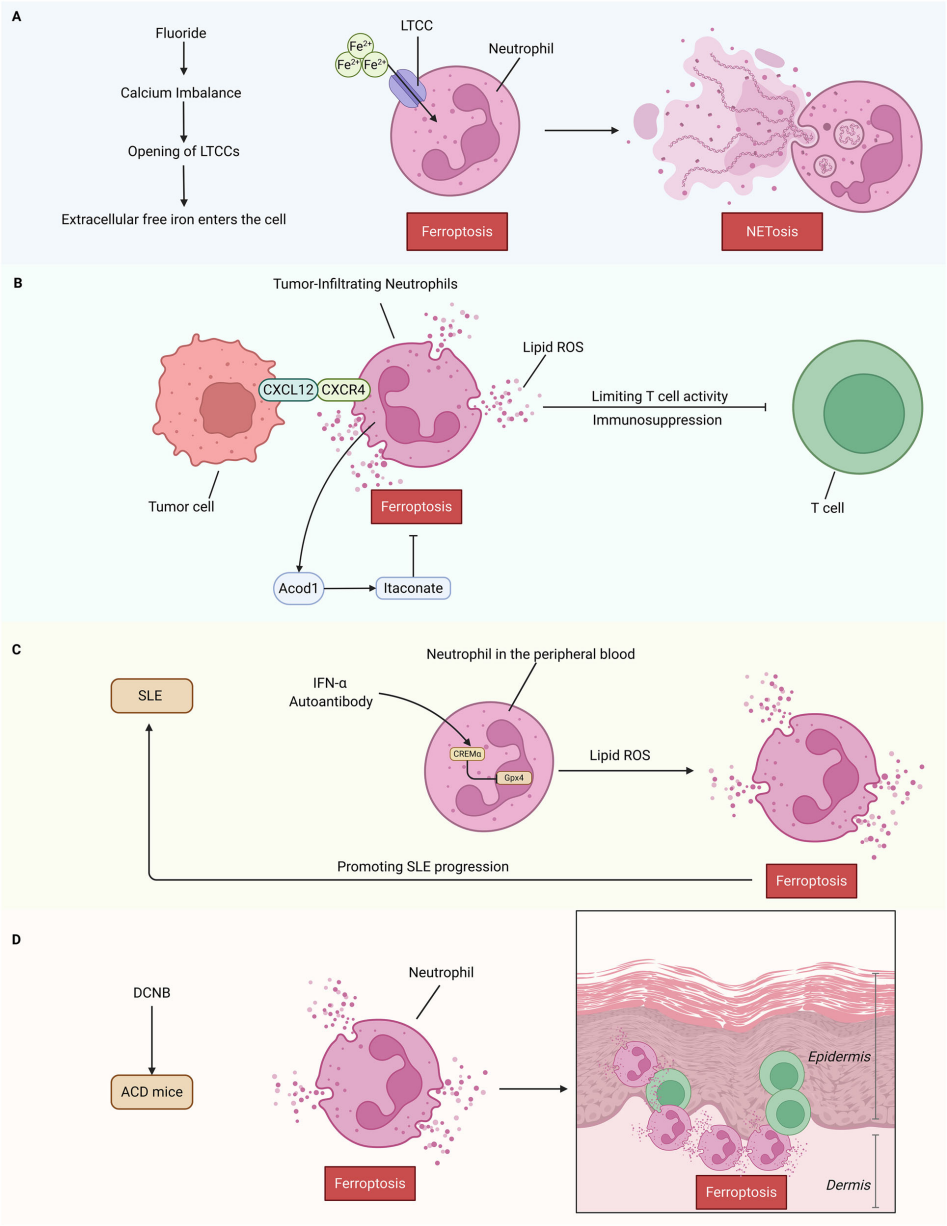
Neutrophil Ferroptosis. Fluorine can induce ferroptosis and inflammatory responses in the brains of rats via Neutrophil Extracellular Traps (NETs). By disrupting calcium homeostasis in neutrophils, fluorine causes the opening of L-type Calcium Channel (LTCC). Extracellular free iron enters the cell through the open LTCC, leading to neutrophil ferroptosis and the release of NETs (**A**). In hepatoma cells, C-X-C chemokine receptor type 4 (CXCR4) is highly expressed in Tumor-Infiltrating Neutrophils (TIN), and its ligand C-X-C motif Chemokine Ligand 12 (CXCL12) is expressed at much higher levels in the tumor than in the surrounding tissue. TIN exhibits ferroptosis and immunosuppressive characteristics, releasing oxidized lipids and restricting T cell activity. However, another study shows that Aconitate decarboxylase 1 (Acod1) is the most upregulated metabolic enzyme in human and mouse TIN. Acod1, produce itaconic acid, and mediate Nuclear factor erythroid 2-related factor 2 (Nrf2)-dependent ferroptosis resistance allowing TIN to survive in the Tumor Microenvironment (TME) (**B**). In patients with Systemic Lupus Erythematosus (SLE), neutrophils undergo ferroptosis, leading to the disruption of immune tolerance in SLE. Mechanistically, autoantibodies present in the serum and Interferon-alpha (IFN-α) enhance the binding of transcriptional repressor cAMP-responsive element modulator alpha (CREMα) to the promoter of Glutathione peroxidase 4 (GPX4) by increasing the transcription of ferroptosis-inducing factor, inducing neutrophil ferroptosis, thereby inhibiting the expression of GPX4 and increasing lipid peroxidation activity (**C**). In contact dermatitis induced by Dinitrochlorobenzene (DCNB) in Allergic Contact Dermatitis (ACD) mice, neutrophils and CD8 + T cells undergo ferroptosis, with ferroptosis being more pronounced in the dermis than in the epidermis (**D**). Created in https://BioRender.com.

Fluoride can induce ferroptosis and inflammatory responses in neutrophils in the rat brain, which exacerbate neuronal inflammation [45] (Fig. 4). The tumor microenvironment (TME) is the local biological environment of solid tumors and is an important target for cancer therapy. In the TME of gastric cancer, tumor-infiltrating neutrophils (TINs) spontaneously undergo ferroptosis, releasing oxidized lipids that limit T cell activity [46]. In hepatoblastoma, C-X-C chemokine receptor type 4 (CXCR4) is highly expressed in TINs, while its ligand C-X-C motif chemokine ligand (CXCL) 12 is expressed at much higher levels in the tumor than in the surrounding tissue. TINs exhibit characteristics of ferroptosis and immune suppression, releasing oxidized lipids and limiting T cell activity [47]. However, another study indicates that TINs are resistant to ferroptosis. TINs produce itaconate, which prevents ferroptosis and allows TINs to survive in the TME [48]. Aconitate decarboxylase 1 (Acod1) is the most upregulated metabolic enzyme in human and mouse TINs. The Granulocyte-Macrophage Colony-Stimulating Factor (GM-CSF-JAK) /STAT5- CCAAT/enhancer-binding protein β(C/EBPβ) pathway can activate Acod1 to produce itaconate, mediating Nuclear factor erythroid 2–related factor 2 (Nrf2)-dependent ferroptosis resistance and maintaining TIN persistence [48]. Moreover, in chemotherapy-resistant breast cancer, IL-1β + CXCL3 + CD4 + T cells mediate neutrophil ferroptosis, suppressing anti-tumor immunity. This suggests that interfering with intercellular crosstalk may be a strategy to reverse chemotherapy resistance [49] (Fig. 4). Current research has not yet fully elucidated why TINs exhibit both susceptibility and resistance to ferroptosis across different tumor types or even within the same tumor model. Based on existing evidence, the following interconnected mechanisms can be summarized. Tumor type-specific variations in metabolic enzyme expression, exemplified by Acod1 upregulation in certain malignancies, and activation states of key signaling pathways, such as GM − CSF-JAK signaling may affect susceptibility of TINs ferroptosis. Furthermore, heterogeneity in cytokine/chemokine profiles within the TME also matters. These factors act in concert with immune cell crosstalk, particularly regulatory interactions between T cell subsets and neutrophils to determine whether TINs undergo ferroptosis.

Furthermore, neutrophils from lupus-prone mice or Systemic Lupus Erythematosus (SLE) patients undergo ferroptosis, which leads to the disruption of immune tolerance in SLE. Mechanistically, autoantibodies and Interferon (IFN)-α present in the serum enhance the binding of the transcriptional repressor cAMP-responsive element modulator alpha (CREMα) to the GPX4 promoter, inducing neutrophil ferroptosis. This process suppresses GPX4 expression and increases lipid ROS. Treatment with ferroptosis inhibitors significantly reduces disease severity in lupus-prone mice, highlighting the important role of neutrophil ferroptosis in the pathogenesis of SLE [50]. In a mouse model of allergic contact dermatitis (ACD) induced by dinitrochlorobenzene (DNCB), both neutrophils and CD8 + T cells undergo ferroptosis, with more pronounced ferroptosis in the dermis than in the epidermis. Ferrostatin-1 inhibits ferroptosis in neutrophils and CD8 + T cells in ACD mice, thereby alleviating skin damage [51]. The Forkhead box protein O1(FOXO1)- TfR mechanism leads to ferroptosis in neutrophils with high FOXO1 expression following traumatic brain injury. This also disrupts iron homeostasis in oligodendrocytes [52]. In sepsis-associated acute lung injury, transcription of Myosin Light Chain Kinase mediated by the serum response factor (SRF) can prevent ferroptosis in polymorphonuclear neutrophils [53]. Neutrophil ferroptosis has been identified as a critical driver of inflammation and tissue damage in SLE, contact dermatitis, traumatic brain injury, and sepsis-related acute lung injury, and its inhibition markedly alleviates disease severity.

Eosinophils and basophils are members of the granulocyte family, sharing a common lineage with neutrophils, and are important components of the immune system [54]. The cAMP response element-binding protein (CREB) plays a significant role in the transcription of pro-inflammatory genes, but its role in childhood asthma is not yet clear. Studies have found that CREB gene knockdown can significantly promote ferroptosis in eosinophils, enhancing dexamethasone-induced eosinophil death [55]. Currently, there is no direct evidence of basophils undergoing ferroptosis.

### Macrophages

Macrophages are phagocytic immune cells that regulate metabolism and immune responses. They play a crucial role in iron transport by recycling iron from hemoglobin degradation. Macrophages engulf red blood cells, breaking down hemoglobin into heme, which is further degraded into biliverdin, Carbon monoxide, and iron [56].

Macrophages exist in two polarization states: M1 (pro-inflammatory) and M2 (anti-inflammatory). M1 macrophages, induced by Lipopolysaccharide (LPS) and IFN-γ, produce ROS, releasing cytokines like IL-6, IL-1β, and Tumor Necrosis Factor-α (TNF-α). M2 macrophages have low ROS levels and promote tissue repair [57].

When excessive erythrocyte phagocytosis or ferritin degradation triggered by Mycobacterium tuberculosis infection leads to increased iron content [58], iron balance mechanism may be disrupted, resulting in cellular ferroptosis. Macrophage ferroptosis was first described in hemochromatosis, where iron citrate overload can induce ferroptosis in primary mouse hepatocytes and bone marrow-derived macrophages, accompanied by upregulation of Solute Carrier Family 7 member 11(SLC7A11), increased ROS, and increased nuclear Nrf2 [59]. In a mouse transfusion model, after rapid uptake of a large number of erythrocytes, the increase in HO-1 is not sufficient to counteract the large amount of erythrocyte phagocytosis, leading to macrophage ferroptosis [60]. ACSL4 induces ferroptosis in inflammatory macrophages, exacerbating fibrosis progression in a systemic sclerosis model [61]. In clinical settings of infection, blood transfusion, or hereditary iron overload, monitoring and modulating macrophage iron load may serve as a novel strategy for interrupting the ferroptosis–inflammation–fibrosis cascade and improving patient outcomes.

#### The ratio of M1 to M2 phenotypes

Compared with M2 cells, activated M1 macrophages are less sensitive to drug-induced ferroptosis, a difference that has been shown to depend on the production of nitric oxide (NO•). The NO• produced by inducible Nitric Oxide Synthase (iNOS) is a reactive radical that can interact with other radicals, including lipid radicals. The iNOS-deficient M2 phenotype exhibits high sensitivity to iron-containing stimuli, a sensitivity that is completely eliminated by the exogenous addition of NO• donors [62].

**Fig. 5:**
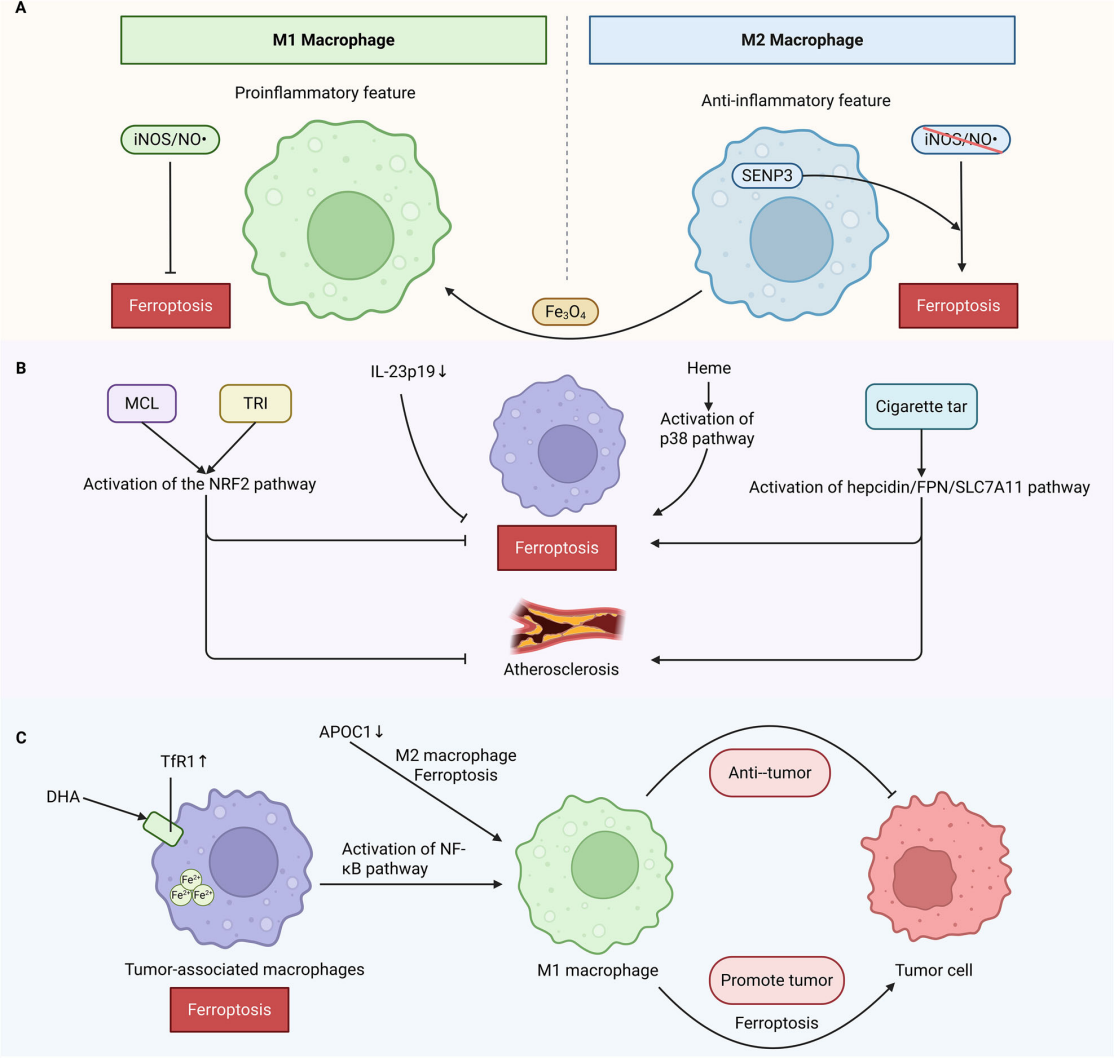
Macrophage ferroptosis. Compared with M2 macrophages, activated M1 macrophages are less sensitive to drug-induced ferroptosis, a difference that has been shown to depend on the production of Nitric Oxide (NO). NO• produced by Inducible Nitric Oxide Synthase (iNOS) is a reactive free radical capable of interacting with other free radicals (including lipid radicals). Sentrin/SUMO-specific Protease 3 (SENP3) sensitizes macrophages to RSL3-induced ferroptosis and SENP3 can promote ferroptosis in M2 macrophages. Fe_3_O_4_ nanoparticles can reduce the viability of macrophages and induce polarization of macrophages towards the M1 phenotype after treatment (**A**). Excessive lipids can also promote ferroptosis in macrophages, such as in the case of atherosclerotic plaques. Micheliolid (MCL) activates the Nuclear Factor Kappa-light-chain-enhancer of Activated B cells (NF-κB) pathway, inhibiting ferroptosis in macrophages, and suppressing atherosclerosis. Tricetin (TRI) is a potential therapeutic agent for atherosclerosis, activating the Nuclear Factor Erythroid 2-related Factor 2 (Nrf2) pathway to inhibit ferroptosis and oxidative stress in macrophages. Cigarette tar significantly promotes the formation of lipid-rich plaques in atherosclerotic lesions, causing severe iron overload and lipid peroxidation, with hemoglobin-induced ferroptosis in macrophages promoting the activation of the p38 Mitogen-activated Protein Kinase (p38 MAPK) pathway and the expression of Matrix Metalloproteinase 2/9 (MMP2/9). Knocking out Interleukin-23 p19 (IL-23p19) can reduce ferroptosis in macrophages (**B**). Tumor-associated Macrophages (TAM) with M2 phenotype contribute to tumor immune suppression. Therefore, repolarization of TAM towards the M1 phenotype and induction of ferroptosis have become therapeutic targets. Conversely, if M1-type macrophages undergo ferroptosis, it can accelerate tumor progression (**C**). Created in https://BioRender.com.

M2 macrophages are more susceptible to ferroptosis than M1 macrophages, and this susceptibility is associated with the Extracellular signal-regulated kinase (ERK)-Cytosolic phospholipase A2 (cPLA2)-ACSL4-mediated activation of the arachidonic acid metabolic pathway [63]. Sentrin/SUMO-specific protease 3(SENP3), a redox-sensitive protease, plays an important role in macrophage function. SENP3 sensitizes macrophages to RSL3-induced ferroptosis and can promote ferroptosis in M2 macrophages, thereby reducing the proportion of M2 macrophages [64]. Iron oxide (Fe_3_O_4_) nanoparticles can reduce macrophage viability after 48 h of treatment and induce macrophage polarization towards the M1 phenotype, as well as induce ferroptosis in macrophages [65] **(**Fig. 5**)**.

#### Atherosclerosis and cardiac remodeling

Excessive lipids can also promote ferroptosis in macrophages, for example, in the case of atherosclerotic plaques. Micheliolide inhibits macrophage ferroptosis and atherosclerosis by activating the Nrf2 pathway [66]. Tricetin, a potential therapeutic agent for atherosclerosis, provides a new strategy for slowing disease progression by activating the Nrf2 pathway and inhibiting macrophage ferroptosis and oxidative stress [67]. Conversely, male Apolipoprotein E-deficient mice fed a high-fat diet and intraperitoneally injected with cigarette tar for 16 weeks showed that cigarette tar significantly promoted the formation of lipid-rich plaques in atherosclerotic lesions, causing severe iron overload and lipid peroxidation. This suggests that cigarette tar induces macrophage ferroptosis via the Nuclear Factor kappa-light-chain-enhancer of activated B cells (NF-κB)-activated hepcidin/FPN/SLC7A11 pathway, thereby promoting the progression of atherosclerosis [68]. In addition, heme-induced macrophage ferroptosis promotes the activation of the p38 mitogen-activated protein kinase (p38 MAPK) pathway and the overexpression of Matrix Metalloproteinase (MMP) 2/9, playing a key role in increasing the susceptibility of hemorrhagic plaques. These findings provide insights into the pathogenesis of hemorrhagic plaques, and targeting macrophage ferroptosis may be a promising strategy for stabilizing vulnerable plaques [62]. IL-23p19 may be a potential target for the prevention and treatment of cardiac remodeling. Knocking out IL-23p19 reduces macrophage ferroptosis and improves the cardiac remodeling process [69] **(**Fig. 5**)**. Macrophage ferroptosis has emerged as a pivotal node that links lipid metabolism, inflammatory signaling, and plaque vulnerability in the cardiovascular field. The aforementioned studies provide multiple therapeutic interfaces.

#### Tumor

The M2 phenotype of tumor-associated macrophages (TAMs) contributes to tumor immunosuppression. Consequently, repolarizing TAMs toward the M1 phenotype and inducing ferroptosis have emerged as therapeutic strategies [57]. Dihydroartemisinin (DHA) elevates intracellular iron levels by upregulating TfR1 and triggers ferroptosis in TAMs within lung cancer by inhibiting GPX4. Lipid peroxidation induces Deoxyribonucleic Acid (DNA) damage response, further activating NF-κB to promote macrophage polarization toward the M1 phenotype [70]. Apolipoprotein C-1 (APOC1) was found to be overexpressed in TAMs of human hepatocellular carcinoma tissues. Suppression of APOC1 reverses macrophage polarization from the M2 to M1 phenotype through ferroptosis [71]. SLC7A11-mediated ferroptosis in macrophages significantly upregulates Programmed Death-Ligand 1(PD-L1) expression in macrophages, thereby enhancing the antitumor efficacy of anti-PD-L1 therapy [72]. Conversely, microRNA-142-3p promotes ferroptosis in Hepatitis B Virus -infected M1 macrophages via Solute Carrier Family 3 Member 2 (SLC3A2), affecting GSH and Fe^2+^ production, which accelerates the progression of hepatocellular carcinoma [73] **(**Fig. 5**)**. Thus, the cell-type selectivity of ferroptosis between M2 and M1 macrophages remains undefined. Identical ferroptosis can yield diametrically opposite outcomes, either immune-enhancing or immunosuppressive, depending on the TAM subset, viral infection status, and underlying metabolic milieu.

#### Inflammation

M2 macrophages in the rheumatoid arthritis (RA) synovial microenvironment are particularly susceptible to ferroptosis, where the HMGB1/ Toll-like Receptor 4 (TLR4)/STAT3 axis plays a pivotal role in exacerbating synovial inflammation [74]. Clostridium perfringens beta-1 toxin (CPB1), a lethal toxin responsible for necrotic enteritis, may induce macrophage ferroptosis through sustained intracellular calpain activation and excessive oxidative stress. Inhibition of calpain reduces oxidative stress and mitigates CPB1-induced macrophage ferroptosis [65]. Silica exposure triggers ferroptosis in macrophages, accompanied by enhanced inflammatory responses. This process involves activation of the Wingless-type MMTV integration site family, member 5a (Wnt5a)/Ca^2+^ signaling pathway, endoplasmic reticulum stress, and mitochondrial redox imbalance [75]. Whether in the HMGB1/TLR4/STAT3 axis of the RA synovium or the Wnt5a/Ca²⁺ signaling pathway, a shared knowledge gap persists. Is ferroptosis the spark that ignites inflammation or merely a collateral form of cell death that accompanies it? Current evidence indicates that ferroptosis is not a terminal epiphenomenon but rather an active “signal amplifier” of the inflammatory response.

#### Spinal cord injury/pulmonary injury/hepatic injury

In a murine model of spinal cord injury (SCI), the small-molecule inhibitor CA-074-me significantly reduced macrophage ferroptosis by attenuating lipid peroxidation and mitochondrial dysfunction. Treatment with CA-074-me not only suppressed macrophage ferroptosis but also promoted M2 macrophage polarization, ultimately enhancing functional recovery post-SCI [76].

In sepsis-induced acute lung injury (ALI), uridine demonstrated protective effects by activating the Nrf2 signaling pathway to inhibit macrophage ferroptosis [77]. Additionally, Triggering Receptor Expressed on Myeloid cells 2 (TREM2) was found to be downregulated in LPS-treated macrophages and in ALI mouse models. Mechanistically, TREM2 deficiency exacerbated ferroptosis via p38 MAPK and STAT6 signaling pathways, while TREM2 overexpression conferred protection against LPS-induced ALI by suppressing macrophage ferroptosis [78].

Single-cell RNA sequencing of murine macrophages identified a distinct and highly sensitive Kupffer cell subset (Clec4F + /CD206 + ) that undergoes Heme Oxygenase-1(HMOX-1) -mediated ferroptosis. This process triggers IL-1β release, contributing to heat stroke-induced liver injury [79].

### Dendritic cells

Dendritic cells (DCs) serve as central players in the initiation, regulation, and maintenance of immune responses. Immature DCs exhibit robust antigen-engulfing capacity and differentiate into mature DCs upon antigen stimulation. DCs interact with T cells to stimulate immune responses, thereby being recognized as the most potent antigen-presenting cells. DCs can recognize tumor-specific antigens and activate CD8 + T cells via T cell receptor (TCR) signaling, leading to the release of IFN-γ by activated T cells, which subsequently induces ferroptosis in cancer cells [80].

Conversely, ferroptosis in DCs disrupts the CD8 + T cell activation pathway, impairing IFN-γ production by CD8 + T cells and suppressing anticancer immunity. Han et al. demonstrated that the nuclear receptor Peroxisome Proliferator-Activated Receptor Gamma (PPARγ), involved in lipid metabolism regulation, promotes RSL3-induced ferroptosis in DCs [81]. Genetic ablation of PPARγ restores DC maturation and function, enabling cytotoxic T cell activation through signal transduction and enhancing CD8 + T cell-mediated antitumor immunity. In an orthotopic glioblastoma rat model, suppressing Ras-related protein Rab-27a expression inhibited the secretion of glioblastoma-derived exosomes, reduced lipid accumulation in infiltrating DCs within the brain, and decreased lipid peroxidation levels in mature DCs, thereby suppressing glioblastoma growth [82]. DCs in the TME express high levels of PD-L1. Mechanistically, PD-L1 deficiency leads to increased lipid peroxidation, DC ferroptosis, and suppression of antitumor immune responses [83]. Activation of the endoplasmic reticulum stress response factor X-box binding protein 1 (XBP1) in DCs suppresses antitumor immunity by driving abnormal lipid accumulation. This lipid accumulation impairs DC-T cell interactions, whereas XBP1 silencing in DCs enhances their antitumor functionality and immunostimulatory dendritic cell activity [84]. In summary, DCs can initiate antitumor immunity by inducing ferroptosis in cancer cells via the TCR–IFN-γ axis, yet their own ferroptosis, triggered by PPARγ activation, PD-L1 deficiency, or XBP1-driven lipid accumulation, abrogates antigen presentation and suppresses CD8⁺ T cell activity. Targeting DC ferroptosis or reprogramming their lipid metabolism has thus emerged as a novel strategy to restore antitumor immunity.

### T lymphocytes

In addition to promoting tumor cell death, ferroptosis also exerts robust immunosuppressive effects within the TME by modulating both innate and adaptive immune responses. Ferroptosis plays a dual role in tumor immunology, influencing either antitumor or pro-tumor functions of immune cells. A deeper understanding of the mechanisms underlying T cell dysfunction in the TME will significantly advance cancer immunotherapy.

#### CD8 + T cell

**Fig. 6:**
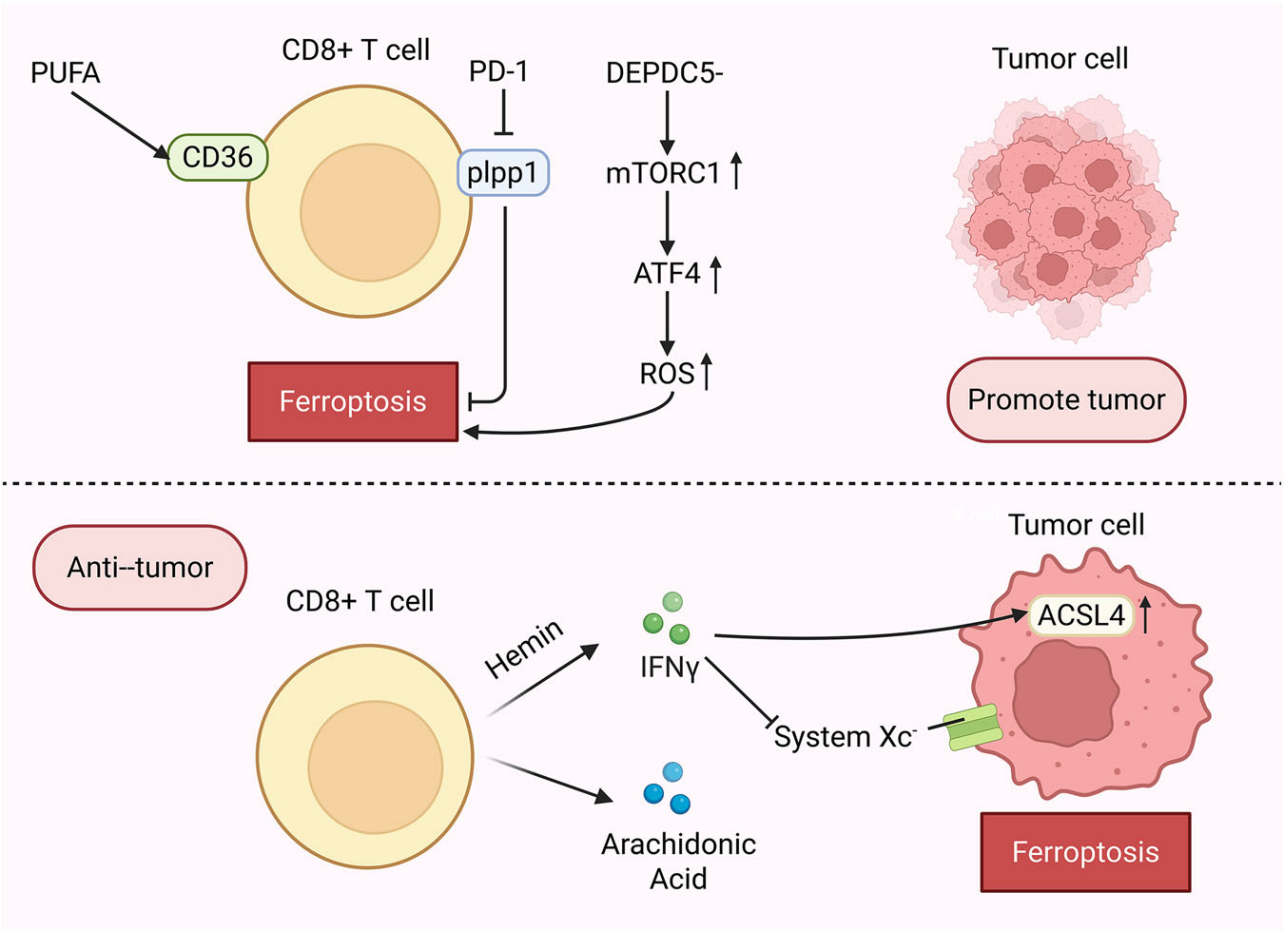
CD8 + T cell related ferroptosis. Under normal conditions, CD8 + T cells secrete Interferon-gamma (IFN-γ), which reduces the expression of System Xc- (Solute Carrier Family 3 Member 2 (SLC3A2) and Solute Carrier Family 7 Member 11 (SLC7A11)), stimulates the expression of Acyl-CoA Synthetase Long Chain Family Member 4 (ACSL4), and alters the lipid profile of tumor cells, thereby exhibiting anti-tumor capabilities. The occurrence of ferroptosis in CD8 + T cells impairs damages their anti-tumor ability. CD36 mediates the uptake of fatty acids by tumor-infiltrating CD8 + T cells in the tumor microenvironment, induces lipid peroxidation and ferroptosis, leading to a decrease in the production of cytotoxic cytokines and compromised anti-tumor ability. CD8 + T cells with defective DEP Domain Containing 5 (DEPDC5) exhibit spontaneous ferroptosis due to high expression of Xanthine Oxidase (XO) and lipid Reactive Oxygen Species (ROS) induced by high Mechanistic Target of Rapamycin Complex 1 (mTORC1)-induced Activating Transcription Factor 4 (ATF4) expression. The Programmed Cell Death Protein 1 (PD-1) signal limits the expression of Phosphatidylinositol Phosphatase 1 (Plpp1), promoting ferroptosis in tumor-infiltrating CD8 + T cells. Created in https://BioRender.com.

CD36-mediated ferroptosis suppresses CD8 + T cell effector function and impairs their antitumor capacity. Elevated CD36 expression in tumor-infiltrating CD8 + T cells is associated with tumor progression and poor survival in both human and murine cancers. CD36 facilitates fatty acid uptake in tumor-infiltrating CD8 + T cells within the TME, inducing lipid peroxidation and ferroptosis, which leads to reduced cytotoxic cytokine production and compromised antitumor activity [85, 86]. Due to the high expression of SLC7A11, tumor cells outcompete T cells for cystine uptake, a mechanism that promotes CD36-mediated CD8 + T cells [87]. Anti-CD274 (PD-L1) immunotherapy triggers IFN-γ release from CD8 + T cells, which suppresses SLC7A11 and induces ferroptosis, thereby enhancing immunotherapy efficacy [88]. Similarly, ACSL4-dependent tumor ferroptosis, induced by the combined effects of IFN-γ and arachidonic acid, also elicits CD8 + T cell-dependent antitumor immunity [89] (Fig. 6).

Hemin enhances CD8 + T cell IFN-γ secretion, and elevated IFN-γ synergizes with hemin to induce tumor cell ferroptosis [90]. Mechanistically, IFN-γ downregulates System Xc⁻, impairing cystine uptake in tumor cells, depleting GSH, and sensitizing tumors to ferroptosis. In the presence of arachidonic acid and other fatty acids, IFN-γ stimulates ACSL4 expression, alters tumor cell lipid metabolism, and promotes ferroptosis [90]. IL-9-secreting CD8 + Tc9 cells (a cytotoxic T lymphocyte subset) activate STAT3, upregulate fatty acid oxidation and mitochondrial activity, and exhibit reduced lipid peroxidation in the TME, thereby resisting tumor- or ROS-induced ferroptosis [91] (Fig. 6).

DEP domain containing 5 (DEPDC5) deficient CD8 + T cells exhibit spontaneous ferroptosis due to mTORC1-induced Activating Transcription Factor 4(ATF4) upregulation, leading to elevated xanthine oxidase activity and lipid ROS accumulation [92]. Programmed cell death protein 1(PD-1) signaling suppresses phospholipid phosphatase 1 (Plpp1), promoting ferroptosis in intratumoral CD8 + T cells. Conversely, PD-1 blockade increases Plpp1 expression, restoring CD8 + T cell antitumor function [93]. CD8 + T cell function is bidirectionally regulated by ferroptosis, tumor cells weaken immunity by competing for cystine and CD36-mediated lipid peroxidation to induce T cell ferroptosis, whereas IFN-γ and related signals reverse this process by driving ferroptosis in tumor cells, thereby enhancing therapeutic efficacy. Additionally, CD8 + T cells are susceptible to ferroptosis regulation by other immune cells in the TME, including TAMs, DCs, Tregs, and myeloid-derived suppressor cells. Together, these factors form a complex network linking CD8 + T cell function, ferroptosis, and cancer immunity **(**Fig. 6**)**.

#### Follicular helper T cells

Follicular helper T (TFH) cells are a specialized subset of CD4 + T cells that primarily support germinal center reactions to generate high-affinity, long-lived humoral immunity. The selenium-GPX4 axis protects TFH cells from ferroptosis and plays a central role in regulating TFH homeostasis [94]. Iron regulates quiescence in naive CD4 + T cells by controlling mitochondrial and cellular metabolism. Iron-overloaded CD4 + T cells fail to induce mitochondrial iron retention, exhibit increased spontaneous proliferation, and display mitochondrial hyperactivation [95].

In patients with acute type A aortic dissection, CD4 + T cells exhibit a hypofunctional phenotype associated with poor clinical outcomes. These cells show enrichment in ferroptosis and lipid metabolism pathways. CD36 is upregulated in CD4 + T cells, and blocking CD4 + T cell CD36 effectively mitigates ferroptosis and T cell hypofunction [96]. Ferroptosis also modulates immune responses against pathogens. For example, selenium supplementation increases GPX4 expression in TFH cells, reduces ferroptosis susceptibility, and enhances antibody responses in influenza-infected mice [94]. Notably, CD4 + T cell ferroptosis, likely mediated by downregulation of the SLC7A11-GSH-GPX4 pathway, has favorable predictive value for the development of post-traumatic sepsis [97]. The selenium–GPX4 axis shields TFH cells from ferroptosis. Similar to CD8 + T cells, CD36 is a key driver. Iron overload or upregulated CD36 precipitates CD4 + T cell ferroptosis and dysfunction, both of which can be reversed by selenium supplementation or CD36 blockade.

#### Regulatory T cells

High-fat diet (HFD) induces ferroptosis in intestinal Tregs, which may serve as a critical initiating step in disrupting immune tolerance and promoting colitis development. Compared to effector T cells, Tregs exhibit a greater reliance on lipid metabolism and preferentially incorporate PUFAs into membrane phospholipids. Consequently, an HFD rich in PUFAs renders Tregs vulnerable to lipid peroxidation and ferroptosis [98].

Notably, IL-1β is upregulated during GPX4-deficient Treg ferroptosis, which subsequently activates DCs and CD8 + T cells, leading to suppressed tumor growth [99]. Trafficking Protein Particle Complex Subunit 1 (TRAPPC1) intrinsically protects naive T cells from ferroptosis, thereby preventing spontaneous autoinflammatory disease in mice. The constitutive expression of TRAPPC1 in naive T cells plays an indispensable role in maintaining T cell homeostasis and averting autoinflammation caused by proinflammatory naive T cell death [100]. High-fat diet-induced ferroptosis in Tregs disrupts intestinal immune tolerance, while GPX4 deficiency or TRAPPC1 insufficiency elicits antitumor effects/inflammation via IL-1β-mediated immune activation or naive T cell death, highlighting the dual role of ferroptosis in T cell homeostasis and disease.

### B lymphocytes

B lymphocytes can be categorized into three main subsets: B1 cells, follicular (Fo) B2 cells, and marginal zone (MZ) B cells. The distinct lipid metabolic profiles between Fo B2 lymphocytes and B1/MZ B lymphocytes underlie their differential responses to GPX4 deficiency. Jonathan Muri et al. demonstrated that GPX4 is indispensable for the development, maintenance, and functional responses of B1 and MZ B cells, whereas it appears nonessential for follicular B2 cell development and function. Mechanistic studies revealed that compared to Fo B2 cells, B1 and MZ B cells exhibit more active lipid metabolism and greater susceptibility to both lipid peroxidation and ferroptosis [101]. Furthermore, Qian Chen et al. provided evidence of B cell ferroptosis in SLE patients and SLE mouse models, suggesting that ferroptosis may regulate B cell differentiation and plasma cell formation, thereby implicating this process in SLE pathogenesis [102]. Future studies should target lipid metabolism and GPX4 regulation in B1/MZ B cells to clarify the ferroptosis–plasma cell axis in autoimmune diseases and develop clinically translatable, subset-specific intervention strategies.

### Natural killer/ innate lymphoid cells

Natural killer (NK) cells are susceptible to ferroptosis, as evidenced by previous studies demonstrating that GPX4-overexpressing NK-92 cell lines were successfully established [103]. As pivotal players in antitumor immunity, human NK cells exhibit functional impairment in the TME due to oxidative stress associated with lipid peroxidation. Activation of the Nrf2 antioxidant pathway restores NK cell metabolism and function, resulting in enhanced antitumor activity in vivo [104].

Unlike T and B lymphocytes, NK cells can nonspecifically eliminate tumor cells and virus-infected cells without prior sensitization. In concert with CD8 + T lymphocytes, NK cells are potent producers of IFN-γ [105]. The combined action of IFN-γ and specific fatty acids within the TME can induce tumor cell ferroptosis. As IFN-γ can be released by multiple immune cells, including T helper 1 cells, NK cells, these IFN-γ-producing immune cells may trigger ferroptosis in lipid-rich microenvironments [89]. While most ferroptosis studies employ synthetic chemical inducers (e.g., RSL3 and erastin), IFN-γ and fatty acids as endogenous ferroptosis inducers provide crucial insights into physiological ferroptosis regulation [106]. NK cells are susceptible to ferroptosis, and GPX4 serves as their key protector; lipid peroxidation in the TME impairs NK cell antitumor function, whereas Nrf2 activation or GPX4 up-regulation restores their cytotoxic activity. Within the TME, IFN-γ and fatty acids act as endogenous ferroptosis inducers that can affect not only tumor cells but also other immune cells, necessitating further investigation.

Group 3 innate lymphoid cells (ILC3s) play essential roles in intestinal pathogen defense and tissue homeostasis. In ulcerative colitis patients, GPX4 is upregulated in intestinal mucosal ILC3s. GPX4 deficiency leads to reduced NKp46 + ILC3 cell numbers, impaired production of IL-22 and IL-17A, and exacerbation of intestinal inflammation in a T cell-independent manner [107].

## Therapeutic potential of ferroptosis in immune regulation

### Novel therapeutic opportunities in tumor immunotherapy

Targeting key molecules in ferroptosis regulatory pathways presents novel opportunities for tumor therapy. TfR1, an established ferroptosis biomarker in vivo, represents a critical node for ferroptosis-based cancer treatment [108]. Several ferroptosis inducers can directly bind to and reduce GPX4 levels or sensitize cancer cell lines to GPX4 inhibition. Notably, FSP1 has emerged as a novel ferroptosis inhibitor, and its specific inhibitor iFSP1 sensitizes certain cancer cell lines to GPX4 suppression [109]. Altretamine for advanced ovarian cancer has been shown to inhibit GPX4 activity and induce intracellular lipid peroxide accumulation [110]. Other compounds like erastin, sulfasalazine, and sorafenib exert their ferroptosis-inducing effects through System Xc^-^ inhibition [111]. While immune cell subsets in the TME are regulated by ferroptosis, current ferroptosis-inducing drugs may non-selectively trigger ferroptosis in both cancer cells and other TME cells. Recent drug screening has identified N6F11, a previously uncharacterized small molecule that selectively degrades GPX4 in cancer cells without affecting DCs, T cells, or NK cells [112]. In melanoma and lung cancer mouse models, mefloquine enhances anti-PD-1 immunotherapy through the IFN-γ-STAT1 ferroptosis pathway [113]. Increasing intracellular iron concentration represents another ferroptosis induction strategy. For instance, modulating iron oxidation levels (via FINO2) [114] can induce tumor cell ferroptosis. However, since iron plays crucial roles in redox regulation of normal cells, systemic administration of iron modulators may cause severe adverse effects (Tables 2,3).

**Table 2:** Novel therapeutic opportunities targeting ferroptosis.

Substance	Mechanism	Application
iFSP1	Sensitizing cancer cell lines to GPX4 inhibition [109]	Anti-tumor therapy
Altretamine	GPX4 activity inhibition induces intracellular lipid peroxide accumulation [111]	Anti-tumor therapy
Withaferin A	GPX4 protein level reduction triggers ferroptosis in neuroblastoma [130]。	Anti-tumor therapy
Erastin /Sulfasalazine/ Sorafenib	System Xc⁻ inhibition mediates ferroptosis induction [111]	Anti-tumor therapy
N6F11	Targeted GPX4 degradation induces ferroptosis in iron-addicted cancer cells [112]	Anti-tumor therapy
FINO2	Iron oxidation state modulation [114]	Anti-tumor therapy
Mefloquine	IFNγ-STAT1-IRF1-LPCAT3 axis drives ferroptosis [113]	Anti-tumor therapy
FeSO_4_	FeSO₄ promotes Staphylococcus aureus ferroptosis via ROS generation and lipid peroxidation [115]	Anti-infective therapy
FOT1	Hepatic iron overload accelerates MASH progression through c-Myc-ACSL4-mediated ferroptosis [118]	Anti-inflammatory therapy
AS-252424	Phosphatidylinositol 3-kinase γ-independent ferroptosis suppression ameliorates acute kidney injury [119]	Anti-inflammatory therapy
GCXXD	ACSL4-mediated ferroptosis inhibition treats ulcerative colitis by restoring intestinal epithelium [120]	Anti-inflammatory therapy
Erastin (IKE)	Synovial fibroblast reduction alleviates arthritis progression [126]	Treatment of Arthritis
liproxstatin-1	Autoantibody production attenuation improves lupus nephritis [102]	Treatment of Lupus

**Table 3:** Novel therapeutic opportunities in immune cell-related therapies targeting ferroptosis.

Substance	Immune cells	Mechanism	Application
IFN-γ and arachidonic acid	CD8 + T cell	ACSL4-dependent tumor ferroptosis induced by the combination of IFN-γ and arachidonic acid triggers CD8 + T cell-dependent antitumor immunity [89]	Anti-tumor therapy
Hemin	CD8 + T cell	Activation of CD8 + T cell IFN-γ secretion promotes hemin-induced tumor cell ferroptosis [90]	Anti-tumor therapy
Dihydroartemisinin	Macrophage	GPX4 inhibition triggers macrophage ferroptosis in lung cancer, polarizing toward M1 phenotype [70]	Anti-tumor therapy
Vitamin E	Regulatory T cell	Attenuating Treg ferroptosis enhances Treg population/function and ameliorates necrotizing enterocolitis [116]	Anti-inflammatory therapy
BCP	Macrophage	Macrophage ferroptosis inhibition alleviates UC murine colitis [121]	Anti-inflammatory therapy
CLF	Macrophage	Enhanced IBD therapy via M2/M1 macrophage ratio modulation coupled with ferroptosis suppression [122]	Anti-inflammatory therapy
FINs	Eosinophil	Eosinophil atypical ferroptosis induction for eosinophilic airway inflammation treatment [123]	Anti-inflammatory therapy
Butachlor	Macrophage	P62 downregulation promotes Keap1-mediated Nrf2 degradation, inducing splenic macrophage ferroptosis [131]	Anti-inflammatory therapy
RvD1	Macrophage	FPR2 immunomodulation reduces macrophage ferroptosis and HMGB1 release in aortitis [124]	Anti-inflammatory therapy
GTP Cyclohydrolase 1	Macrophage	Suppression of LPS-induced macrophage ferroptosis in pneumonia [125]	Anti-inflammatory therapy
Selenium	Granulocyte	Granulocyte oxidative stress marker normalization [50]	Treatment of psoriasis
LPX-1	Macrophage	K/BxN serum-transfer arthritis amelioration with increased M2 macrophage proportion [127]	Treatment of arthritis
CX-5461	B lymphocyte	B cell ferroptosis induction via p53-SLC7A11-ALOX12 axis [129]	Treatment of lupus

### Therapeutic strategies targeting infection and inflammation-related immunity

The induction of bacterial ferroptosis represents a promising strategy for developing novel antibiotics against drug-resistant infections. The emergence of methicillin-resistant Staphylococcus aureus has complicated antibiotic-based hydrogel treatments, prompting development of new antimicrobial materials. FeSO_4_ exhibits bactericidal activity against S. aureus by promoting ferroptosis through enhanced ROS generation and lipid peroxidation, with FeSO_4_-loaded hydrogels demonstrating therapeutic potential in murine keratitis models [115]. In immunoregulation, vitamin E alleviates Treg cell ferroptosis, enhancing their quantity and function to mitigate intestinal damage in necrotizing enterocolitis [116], while high-dose supplementation prevents infection-induced immunodeficiency by protecting GPX4 + T cells [110]. Inhibition of Neutrophil Extracellular Traps (NETs) and enhancement of FUN14 Domain Containing 1 (FUNDC1)-dependent mitophagy attenuate microvascular endothelial ferroptosis in intestinal inflammation [117]. In metabolic-associated steatohepatitis, the iron chelator FOT1 inhibits disease progression by targeting the c-Myc-ACSL4 axis, with serum ferritin serving as a treatment biomarker [118]. The PI3Kγ inhibitor AS-252424 suppresses ferroptosis independently of PI3Kγ, reducing renal inflammation [119]. For ulcerative colitis (UC), Gancao Xiexin decoction (GCXXD) inhibits ACSL4-mediated ferroptosis in intestinal epithelial cells, promoting mucosal repair [120], while β-caryophyllene (BCP) ameliorates colitis by suppressing macrophage ferroptosis [121]. In inflammatory bowel diseases, CaCO_3_-mineralized liposomes (CLF) encapsulating Ferrostatin-1 enhance M2 macrophage polarization while inhibiting ferroptosis [122]. Ferroptosis-inducing agents (FINs) alleviate allergic airway inflammation through iron-dependent but lipid peroxidation-independent eosinophil ferroptosis [123]. Butachlor promotes splenic macrophage ferroptosis via the Sequestosome 1(P62)- Kelch-like ECH-associated protein 1(Keap1)-Nrf2-GPX4 axis, while resolvin D1 attenuates aortic inflammation by reducing macrophage ferroptosis through Formyl Peptide Receptor 2 signaling (FPR2) signaling [124]. Bioinformatic analyses reveal GCH1 inhibits LPS-induced macrophage ferroptosis and M1 polarization [125] (Tables 2–3).

### Novel therapeutic opportunities in autoimmune diseases

Autoimmune diseases encompass a spectrum of disorders caused by aberrant induction of cell death and impaired clearance of self-cells or tissues, including SLE, RA, and multiple sclerosis(MS). Targeting ferroptosis has emerged as a promising therapeutic strategy for mitigating autoimmune pathologies. In RA, synovial fibroblasts exhibit abnormal proliferation dependent on ROS accumulation and lipid peroxidation. In collagen-induced arthritis mouse models, erastin reduces synovial fibroblast numbers and alleviates disease progression [126]. In psoriasis, selenium supplementation normalizes granulocyte oxidative stress markers and significantly improves patients’ clinical outcomes [50]. The ferroptosis inhibitor liproxstatin-1 ameliorates K/BxN serum-transfer-induced arthritis in mice, accompanied by increased M2 macrophage proportions [127]. In MS, ferroptosis promotes T cell activation-induced neurodegeneration. Studies reveal ferroptosis as an early event in experimental autoimmune encephalomyelitis, potentially driven by TCR signaling. These findings implicate ferroptosis as a potential therapeutic target for MS [128]. CX-5461 selectively targets B cells, effectively reducing proportions of total B cell, germinal center B cells, plasma cells in SLE models. Mechanistically, CX-5461 modulates CD36-ACSL4-mediated glycerophospholipid metabolism in B cells, triggers ferroptosis via the p53-SLC7A11- Arachidonate 12-Lipoxygenase (ALOX12) pathway and lowers IgG and anti-dsDNA antibody levels, thus attenuating lupus pathogenesis. CX-5461 thus represents a promising candidate for targeted lupus therapy [129] (Tables 2–3).

## EZH2-mediated epigenetic-ferroptosis-immune Axis

Currently, research on the epigenetics of ferroptosis is still in its infancy; nevertheless, accumulating evidence demonstrates that epigenetic mechanisms, play pivotal roles in regulating ferroptosis. Ferroptosis through epigenetic mechanisms paves the way for innovative treatment approaches in various diseases. Across diverse diseases, robust evidence has documented epigenetic modulation of ferroptosis via mechanisms ranging from DNA methylation [130–132], histone modifications [133–137], chromatin remodeling [138–141] to non-coding RNA (ncRNA)-mediated regulation [142–152] (Table 4).

**Table 4:** Epigenetic regulation in ferroptosis.

Mechanism	Specific mechanism	Target gene	Related disease	Ferroptosis
DNA methylation	Nrf2 Promoter Hypermethylation	Nrf2/GPX4 ↓	Chronic Obstructive Pulmonary Disease	Promotion [130]
DNA methylation	GPX4 Promoter Hypomethylation	GPX4↑	Pan-cancer	Inhibition [131]
DNA methylation	FSP1 Promoter Hypermethylation	FSP1↓	Acute Lymphoblastic Leukemia	Promotion [132]
Histone modification	KDM3B-mediated H3K9 Demethylation	SLC7A11↑	Fibrosarcoma	Inhibition [133]
Histone modification	KDM4A-mediated H3K9 Demethylation	SLC7A11↑	Cervical cancer	Inhibition [134]
Histone modification	G9a-mediated H3K9 Dimethylation	GPX4↓	Multiple Sclerosis	Promotion [135]
Histone modification	SET7/9-mediated H3K4 Trimethylation	RUNX1↑ SIRT6↓	Diabetic Retinopathy	Promotion [136]
Histone modification	JMJD6-mediated Arginine Demethylation	METTL14↓ SLC3A2↑	Lung cancer	Inhibition [137]
Chromatin remodeling	ARID1A loss	GPX4 ↓	Colorectal cancer	Promotion [138]
Chromatin remodeling	SMARCC1	FLOT1↑	Lung cancer	Inhibition [139]
Chromatin remodeling	LSH Cfp1	Ferroptosis-associated metabolic genes↑	Cancer	Promotion [140]
Chromatin remodeling	LSH	CYP24A1 ↓	Colorectal cancer	Promotion [141]
Non-coding RNAs	microRNA-125b-5p	SLC1A5↑	Head and neck squamous cell carcinoma	Inhibition [142]
Non-coding RNAs	microRNA-129-5p	ACSL4↓	Inflammatory Bowel Disease	Inhibition [143]
Non-coding RNAs	Long Non-Coding RNA OTUD6B-AS1	GPX4↑ SLC7A11↑	Breast cancer	Inhibition [144]
Non-coding RNAs	Long Non-Coding RNA LINC02266	ACSL4↓	Gastric cancer	Inhibition [145]
Non-coding RNAs	Long Non-Coding RNA CBSLR	CBS↓	Gastric cancer	Inhibition [146]
Non-coding RNAs	Long Non-Coding RNA HMG	p53↓ SLC7A11↑	Colorectal Cancer	Inhibition [147]
Non-coding RNAs	Long Non-Coding RNA PMAN	SLC7A11↑	Gastric cancer	Inhibition [148]
Non-coding RNAs	Long Non-Coding RNA RGMB-AS1	HMOX1↑	Lung cancer	Promotion [149]
Non-coding RNAs	Long Non-Coding RNA PVT1	GPX4↓	Gastric cancer	Promotion [150]
Non-coding RNAs	Circular RNA Forkhead Box P1	PD-L1-mediated antioxidant signaling↓	Gallbladder carcinoma	Promotion [151]
Non-coding RNAs	Circular RNA PIAS1 encoding a 108-amino-acid protein	SLC7A11↑ GPX4↑	Melanoma	Inhibition [152]

Note: An upward arrow indicates increased expression levels, while a downward arrow signifies decreased expression levels.

Enhancer of zeste homolog 2 (EZH2), the catalytic subunit of the Polycomb Repressive Complex 2 (PRC2) complex, catalyzes the mono-, di-, and trimethylation of lysine 27 on histone H3 (H3K27me3). EZH2 serves as a pivotal node linking epigenetic regulation, tumorigenesis, immune control, and ferroptosis. First, EZH2 serves as the sole catalytic subunit of the PRC2 complex, and its product H3K27me3 is one of the strongest transcriptional silencing signals in the mammalian genome. EZH2 regulates ferroptosis by modulating genes related to iron death, such as ACSL4, SLC7A11, Ubiquitin-Specific Protease 10 (USP10), TFR2, and Atonal bHLH Transcription Factor 8 (ATOH8) [153–157]. Second, EZH2 indirectly participates in sensing oxidative stress. Hypoxia inhibits ferroptosis by suppressing KDM6A (Lysine Demethylase 6A) reshaping cellular phospholipid metabolic profiles. Conversely, inhibiting EZH2 (antagonistic to the function of KDM6A) restores ferroptosis sensitivity as a therapeutic strategy [158].

Additionally, EZH2 simultaneously controls both ferroptosis and immune cell function. It is highly expressed in immune cells, especially those undergoing active proliferation, and orchestrates their development, differentiation, and effector functions [159]. EZH2 maintains the stability of functional T-cell subsets like Treg and TFH, suppresses NK cell maturation and cytotoxicity, inhibits antigen presentation major histocompatibility complex I/II and promotes immunosuppressive TAM polarization [159, 160].

Notably, the EZH2-YY1 axis is a central hub in cancer epigenetic regulation. Targeting the EZH2-YY1 interaction can reverse tumor suppressor gene silencing, restore immune surveillance, and demonstrate broad-spectrum anticancer potential [161]. Studies show that downregulating EZH2 activates Nuclear Receptor Coactivator 4 (NCOA4) to trigger ferroptosis in cancer and enhances antitumor immunity by promoting CD8 + T cell infiltration into tumor tissues [162].

The clinical development of EZH2 inhibitors thus offers novel therapeutic strategies for a broad spectrum of diseases, including cancer, inflammatory disorders, and autoimmune diseases [159]. Targeting EZH2 represents a potential strategy to combine with existing immunotherapies for cancer. EZH2 inhibition disrupts immune evasion through multiple mechanisms, restores the tumor-immune cycle, and enhances the efficacy of immune checkpoint inhibitors [160]. For example, EZH2 inhibitors (e.g., GSK126, Tazemetostat) have entered clinical trials. In triple-negative breast cancer, Tazemetostat enhances HER2-targeted therapy and immunotherapy [163]. After EZH2 inhibitor treatment, tumor cells undergo significant lipid metabolic reprogramming [164], activating the SLC7A11/GPX4 antioxidant axis and upregulating lipid/cholesterol synthesis-related genes (e.g., Stearoyl-CoA Desaturase 1, SCD1), thereby resisting ferroptosis and limiting the efficacy of EZH2 inhibitors. Combining GPX4 inhibitors (e.g., RSL3) with EZH2 inhibitors markedly enhances cellular sensitivity to EZH2 inhibition [165]. Targeting lipid metabolism (e.g,. SCD1 inhibitors) [166] can also overcome resistance to EZH2 inhibitors, providing new therapeutic opportunities [167].

In the Experimental Autoimmune Encephalomyelitis model, microRNA-367-3p inhibits EZH2 via direct targeting, leading to upregulated SLC7A11 expression, GPX4 activation, and ferroptosis suppression [168]. The HMGA1/EZH2/STAT3/GPX4 axis modulates TFH cell ferroptosis to influence SLE progression [169]. GSK126, as an EZH2 inhibitor, significantly attenuates atherosclerosis progression by upregulating ATP-Binding Cassette Transporter A1 [170]. EZH2-mediated STAT3 methylation regulates GPX4 expression and activates ferroptosis pathways, offering novel insights and therapeutic targets for pulpitis treatment [171].

In summary, EZH2 is not only a core epigenetic enzyme in ferroptosis but also plays multifaceted roles in immune responses. Its pivotal position in the ferroptosis-immunity-epigenetic network underscores its unique therapeutic potential.

## Conclusions and perspectives

This paper comprehensively explores the intricate mechanisms of iron metabolism and the principal pathways of ferroptosis, providing an in-depth analysis of the role of iron metabolism in organisms. The review examines ferroptosis in immune cells, discussing how ferroptosis influences immune cell function and revealing its critical role in regulating immune responses. Different immune cells may rely on distinct ferroptosis pathways, leading to varying susceptibility to ferroptosis. Although some progress has been made in studying ferroptosis in immune cells, the specificity of different immune cell types and their complex interactions form a sophisticated regulatory network of ferroptosis. Thus, further in-depth research is needed in the future. This review also summarizes the latest advances in drugs and small molecules targeting ferroptosis in a growing number of immune-related diseases, including cancer, infections, inflammation, and autoimmune diseases. More clinical therapeutics are expected to be developed in the future. Based on important role of EZH2, future research focuses on the integrated regulatory mechanisms between epigenetics and ferroptosis. For example, advancing clinical trials of combination therapeutic strategies utilizing EZH2 inhibitors and ferroptosis inducers to validate their synergistic efficacy in cancer treatment, or developing lipid metabolism-based biomarkers e.g., SCD1 expression level for identifying EZH2 inhibitor-sensitive patient populations. Current research on the epigenetic regulation of ferroptosis has predominantly focused on cancer, while investigations into other disease models remain largely unexplored. Future studies should expand into novel areas such as autoimmune disorders and infectious/inflammatory diseases to facilitate the translation of fundamental discoveries into clinical applications.

## Data Availability

The datasets generated during and/or analyzed during the current study are available from the corresponding author on reasonable request.
